# Social system design methodology for transitioning to a new social structure – a holistic urban living lab approach to the well-being city

**DOI:** 10.3389/fsoc.2023.1201504

**Published:** 2023-10-24

**Authors:** Atsunobu Kimura, Hisashi Haraguchi, Yutaka Yamauchi, Katsuta Matsuura

**Affiliations:** ^1^Co-Designing Institute for Polyphonic Society, Osaka, Osaka, Japan; ^2^Centre for Person-Centred Ningen, Omuta, Fukuoka, Japan

**Keywords:** Living Labs, Sustainability Transition, well-being, smart city, participatory design, co-creation approach, design practice, systemic design

## Abstract

In recent years, along with the rise of new technologies such as AI, IoT, and Bigdata, there has been much discussion replacing entire cities with smart cities. These discussions have given rise to questions about what kind of society should be realized, and keywords such as well-being and sustainability are attracting attention. In this context, how concretely can we transform our current cities into new social structures? Social system design methodology is, in this paper, intend to achieve a comprehensive transition to a new social system, rather than overcoming individual social problems. In Japan, approaches to transforming society, such as elections and social activism, are not fully functional. Transitioning to a new social structure requires critiques from inside together with the presentation of concrete activities. We propose a systematized social system design methodology that aims at a principled transition; it is based on analyses of a series of practices developed in Omuta City, Fukuoka Prefecture in Japan. The methodology proposes a new way of perceiving social systems, practitioner attitude, and a practical design process. It also suggests that existing social system concepts create fundamental problems that lead to discomfort for practitioners, that clarifying existing concepts through policy background analysis can lead to a new view of social system concepts, and that bottom-up practices that realize these new concepts can begin to transform social systems. In order to confirm the versatility of this methodology, two case studies involving care prevention and the work of persons with disabilities are analyzed.

## 1. Introduction

The concept of Sustainable Development Goals (SDGs) aims to integrate the three aspects of society, economy and environment, and to encourage diverse stakeholders, including citizens, governments, and businesses, to transcend sectionalism and work together in a cross-sectional manner toward the meaningful society. As social issues are worsening and becoming more complex, a holistic approach to resolving the social issues is urgently required.

“Social system design” in this paper is also oriented toward the fundamental elimination of problems through a holistic transformation of the social system itself, rather than the piecewise resolution of local problems. Many of today’s social issues are caused by the discrepancy between the existing social system and the reality of our life. Post-event and reactive responses will not lead to a fundamental resolution of the issues or the realization of the future desired. What is important is an approach that perceives the current society as the cause of the problems and aims for its transformation.

## 2. Context

To begin this paper, we first review the weaknesses of conventional approaches in transforming social systems. The social system design discussed in this paper aims to overcome the situations that clearly show the weaknesses of conventional approaches.

In general, political activities are the most common approach to transforming any existing social system. The goal is to translate social ideals into legislation through civil debate in which representatives of citizens discuss and attempt to reach concordance. However, the representative democracy adopted by many democracies only appears to be stable because the class structure of industrial capitalism is balanced against the corresponding mass parties representing social groups, but in post-industrial capitalism, this balance is being lost (Manin, 1997). It has also been said that the unwritten norms of “mutual tolerance” and “organizational self-control,” necessary for democracy to function, are collapsing (Levitsky and Ziblatt, 2018). Thus, the dysfunctionality of representative democracy is being discussed, mainly in developed countries; the same issue has been raised in Japan (Fujii, 2021).

The main alternative to democratic elections is rooted in the diverse needs of citizens. There is a history of citizen movements led by issue groups modifying an existing social system in piecemeal fashion. This approach emphasizes the urgency of position-oriented politics driven by citizen movements; it lies outside traditional parliamentary politics. Civic movements that pursue ownership with minorities as agents emphasize the power relations of dominator / dominated and adopt confrontational actions in order to acquire political resources (Melucci et al., 1989). The assumption is that they can objectify “enemies” external to themselves. However, in the 2000s, in the face of neoliberalism, which has neutralized political antagonisms (Mouffe, 2005) and left-avoiding populism as a situation unique to Japan, it has become impossible to find an easily identifiable enemy, and civic activism is said to have transformed into something that provides a reason for living and a place for people who have difficulty adapting to society (Inaba, 2016).^1^

In other words, both approaches, democratic policy formation and civic activities driven by issue groups, assume a clear-cut adversarial structure, which makes it impossible to establish valid points of contention and resolution. To escape this situation, a new approach quite different from current social structures is required.

## 3. Related studies and research issues

In response to the challenges noted, the search is on for a methodology that overcomes the limitations of the conventional approaches and triggers viable social system transformation; this paper is positioned within this context. Various related studies have attempted to correct the current situation, which has become increasingly pluralistic and complex, and develop architectures that are appropriate for creating rational social structures, rather than tackling the problems with simple oppositional remedies.

Regarding representation, discussions on the various forms of political participation that make democracy function effectively are calling into question the traditional electoral system (Reybrouck, 2016). Some have long advocated “citizen assemblies” that utilize mini-public forums for citizen participation and deliberation (Smith, 2009). Arguments have been made for evaluating the Irish Constitutional Assembly, which experimented with the idea, from the perspective of democratic control over policy making (Donatella della Porta, 2020). However, while these arguments for a more fully democratic system through diversification of the electoral system assume a representative system and rational debates, there is no inherent guarantee that these assumptions will be effective in achieving positive change of the social system. Indeed, they need to be validated empirically. In this respect, this is an argument that awaits further assessment and is beyond the scope of this paper, which is concerned with design methodology.

The following study of group activities for civic change is noteworthy from the viewpoint of the issues raised by this paper. It avoided a reduction to the old oppositional structure of “damage / perpetration” or “individual / government or corporation,” and instead reexamined the civic movements accompanying MINAMATA disease based on the premise that individuals are also embedded in society (Sung, 2003). Based on the interdependency of the individual and society, the perspective of objectifying the cyclically reconstituted social system itself was also emphasized in that paper.^2^ However, the subject of this paper is a practical methodology that takes this cyclicality into account and approaches it in a concrete manner. Along these lines, there is research on Japanese social education theory that discusses the process by which parties to a social issue structurally perceive the issue and transform the community in a learning process called Community Development (Miyazaki, 2019). This is highly suggestive in terms of the internal change of the people involved and the formation of a collective consensus, but it remained within the framework of civil society theory, and so did not include discussions of policy or economics, and did not have the scope needed for holistic social system transformation.

Given this situation, social design^3^ methodologies such as Sustainability Transition (Kohler et al., 2019; Kanger et al., 2020; Markard et al., 2020; Kivimaa et al., 2021) and Urban Living Labs (Cuomo, 2022; Park and Fujii, 2022; Willems, 2022; Afacan, 2023) have attracted attention as concrete practices to ensure participation diversity in support of various parties through collaboration. The research domain of Sustainability Transition; Transition Management details a methodology in which citizens, governments, and businesses co-create a holistic agenda for social system transformation, and apply it in a way that connects it to the specific practices of the agents. However, it lacks a methodology to concretely implement the integrated transformation indicated by the agenda (Roorda et al., 2014), and has yet to fully realize a movement toward this transformation (Kohler et al., 2019). On the other hand, Urban Living Labs is a methodology in which citizens themselves take the initiative and constructively engage with urban stakeholders to solve problems through a design process directed toward sustainable urban transformation (Baccarne et al., 2014). However, a methodology to comprehensively and holistically grasp the complex intertwined elements of the entire city, called Urban Dimensions (Steen and van Bueren, 2017) has yet to be elucidated.

Two points are noteworthy in related research: the first is to take into account the circularity nature of individuals and the social systems, in which the individual is defined by society and the society is defined by the individual. The second is to ensure the diversity of participation through collaboration. However, the nature of the entities and methodologies to realize social system transformation in an integrated manner is a research issue that has yet to be adequately addressed. To contribute to social system transformation, this paper focuses on design methodologies that question the nature of subjects and practices in an integrated manner while overcoming the social conflict structures inherent in the social system through diverse collaborations based on the inner dynamics of circular dependencies of individuals and society (rather than external criticism).

What approach, then, is needed to find such a design methodology? William Gaver, a design and Human-Computer Interaction (HCI) researcher, suggests that the study of “Wicked Problems “(Buchanan, 1992) in design needs to be approached as a “generative discipline.” The engineering analytical method of HCI cannot deal with “Wicked Problems” for which there are no “right” answers, but rather multiple “good” answers. He then stated that design studies’ unique contribution to knowledge is not to move toward generalization, standardization, and theorization based on scientific analysis, but to move toward specialization, diversification, and the generation of artifacts (designs) based on original concepts (Gaver, 2012). Given this perspective, it is a reasonable research approach to carefully describe and analyze the processes of analysis and implementation in each designer’s (practitioner’s) situation.

Therefore, in order to find a methodology for social system design based on the awareness of the aforementioned issues, this paper describes specific practices in Japan in Chapter 4, and attempts to systematize a general-purpose methodology from these practices in Chapter 5. In Chapter 6, we discuss how these methodologies can explain other practices that are oriented toward social system transformation with a similar awareness of the issues.

## 4. Social system design practice in OMUTA city

### 4.1. Emerging social system design practices

OMUTA City contributed greatly to the industrial and economic development of Japan through the mining operations of the MIIKE Coal Mine (1873–1997) by the MEIJI Government and a flourishing coal-chemical complex. However, the population of the city has almost halved from 210,000 in 1959 (the peak of the coal mining era), and the current aging rate is 37.3% (as of October 1, 2022) which is one of the highest in Japan among cities with more than 100,000 people. It is also widely known as an advanced region in terms of dementia care, because the number of people with dementia is increasing in the community, creating a situation in which many people are involved. In Mouffe, 2005, OMUTA City, together with its citizens, issued the “Declaration for Creating a City to Live with People with Dementia”.^4^ The concept of “a town where people can wander around with peace of mind,” which was proposed at that time, was a groundbreaking one. This concept aims to create a town in which people with dementia can live like everyone else in the community, rather than in nursing homes or in communities isolated by gates. Traditionally, the act of wandering and its positioning as a problematic behavior indicated deviation from social customs and triggered treatment and constraint. Given the emergence of similar situations in Japan, OMUTA’s concept is an innovative one that aims to create a town where people with dementia, children, adults, or any other kind of person, are accepted into society.

From the perspective of this paper, this concept and the many practices in OMUTA City that have accompanied it, are the seeds of a social system design practice that finds new meaning for and leads the way to a shift in social systems.

### 4.2. Establishment of an organization for citizens to think and act for the entire city (2019)

It is clear that the activities traditionally proscribed by the issues of “dementia” and “older adult” make it difficult to redesign the entire social system. This is because it is impossible to take account of the social issues that arise in various parts of the community. This is due to the fact that the silo structure of local government limited the areas that could be covered by individual policies. Therefore, the OMUTA Future Co-Creation Center named Center for Person-Centered Ningen, Omuta (hereinafter referred to as “PONI PONI” using the nickname of the organization.) was established in collaboration with the public and private sectors as an “organization that is both independent and embedded” in the existing social system; it crosses vertical divisions in sectors and domains, with a core based on a new deeper concept related to dementia care (Kimura et al., 2019).^5^ PONI PONI was established as a public-private partnership. The founding members included businesses emerging within the community, those who had been involved in OMUTA’s urban development from outside the community, those who shared the concept and had strengths in policy formation outside the community, and design researchers from companies. It is a team structure that is conscious of the fact that its remit is to design social systems.

### 4.3. National model project of health promotion for the elderly health care (2019)

In parallel with the establishment of PONI PONI, we first focused on “care prevention” in response to the situation in OMUTA City, and developed solutions in conjunction with OMUTA City, particularly the “Health Promotion Project for the Elderly Health Care” by the Ministry of Health, Labor and Welfare. This was because we believed that it was necessary to seek the effective integration of two different policy areas: “community-based comprehensive care,” which was being promoted in the medical and long-term care fields, and “regional development,” which aimed to correct the concentration of people, money, and resources in Tokyo. The project involved understanding the policy background of each area and engaging in dialog with practitioners within and outside the region. As a result, we discovered a new transition concept: “from guaranteeing the right to exist (Article 25 of the Constitution) to guaranteeing the right to the pursuit of happiness (Article 13 of the Constitution) (Kikuchi, 2019).” This concept organically connects medical and nursing care with local development. The project report also addressed the Living Labs, which create collaboration between local players and outside companies to solve social issues, and envisions a specific approach for involving companies outside the region.

### 4.4. WAKU WAKU Life Salon (2019)

**Figure 1:**
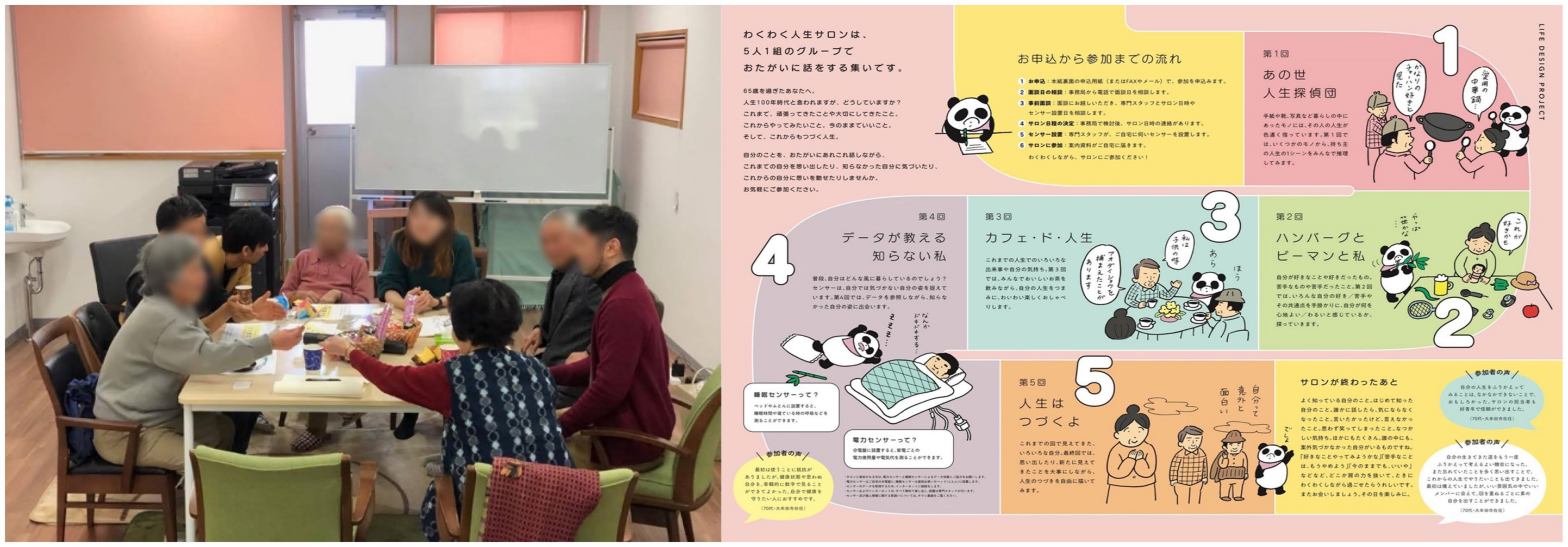
Scene (left) and flyer (right) of WAKU WAKU Life Salon.

Subsequently, as a specific Living Lab practice project, PONI PONI implemented the “WAKU WAKU (This onomatopoeia means that “One’s heart pounded with expectation.”) Life Salon (Figure 1). This project responded to both the needs of local residents and the government to solve problems in OMUTA City and the need of companies to develop new services. In addition, the project embodied Omuta’s new transition concept which is detailed in the aforementioned “Health Promotion Project for Elderly Health Care. Specifically, participants aged 65 or older living in OMUTA City who voluntarily expressed interest in the “WAKU WAKU Life Salon” gathered for a total of five sessions to reflect on their lives to date and their daily lives, and to think about how they could become excited about the remainder of their lives.

For companies, this project was positioned as the search for concepts that would contribute to the development of IoT-based early disease detection services, and to organize UX/UI requirements. The knowledge acquired could be used to launch new commercial entities. At the same time, for residents, the project provided an opportunity for the older adult with limited places to go in the community to regain their motivation. For the government, it was an opportunity to find new measures to deal with matters that could not be approached through the existing long-term care insurance system. The project was designed and managed as a value-added activity in which the three parties involved in the Living Lab overcame their respective challenges.

In this way, we have newly discovered the potential of “dialog that stimulates motivation” in the realization of a new concept through “dialogs” between the older adult and the staff of the WAKU WAKU Life Salon.

### 4.5. Questioning the views of humanity (2020)

After the “WAKU WAKU Life Salon,” a dialog was held with leading practitioners and experts from within the region and beyond to identify a new view of human nature that could comprehensively support corporate service implementation, local practice, and policy development. It became clear that the humanistic view of the “modern subject,”^6^ which is the premise for all institutions and businesses in the modern society and which citizens widely believe should be realized, is no longer compatible with reality and is creating social tension. The dialog also suggested that the identity of the foundational human itself is shared with others and the environment. Furthermore, the phase of identity shifting from role (self-identity) to existence (ego-identity), not through discipline, but through release, and through “dialog,” would stimulate motivation from existence (ego-identity). In other words, a new view of the human being, which is necessary for social system design, was found.

### 4.6. Co-creation of OMUTA city health and welfare comprehensive plan (2021)

**Figure 2:**
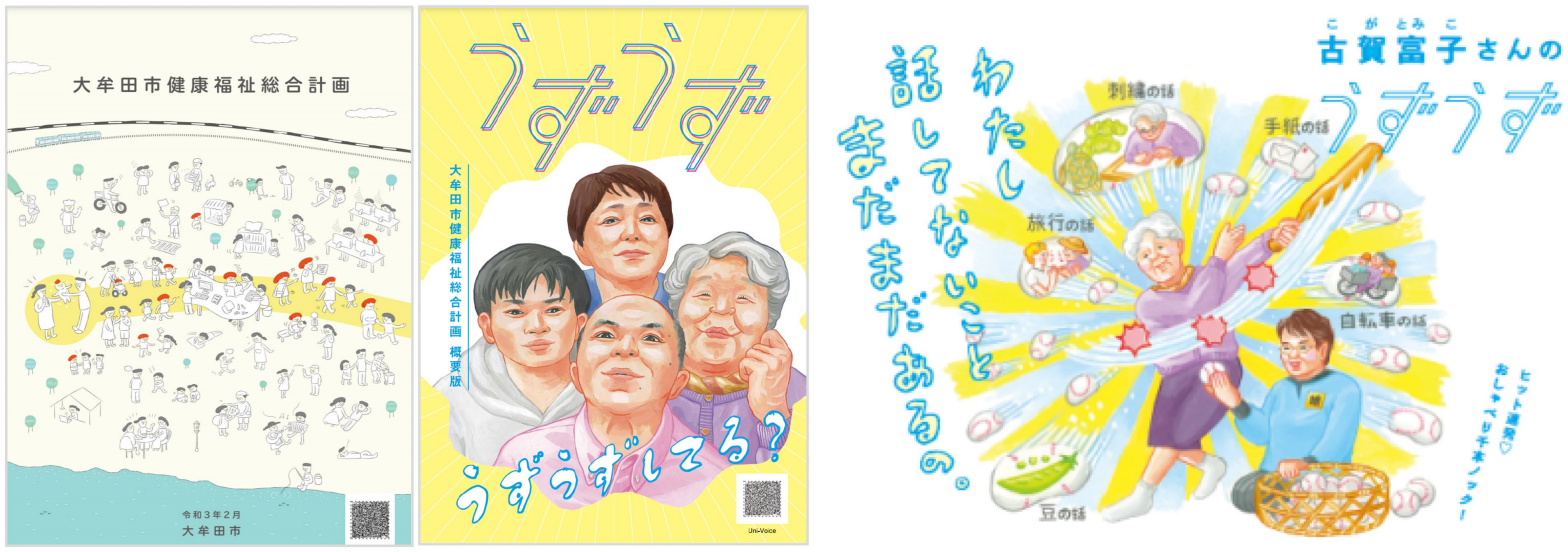
Official booklet (left) and booklet for citizens (center, right).

In order to redesign the entire social system, PONI PONI and OMUTA City collaborated to develop the OMUTA City Health and Welfare Comprehensive Plan (Figure 2), which is a comprehensive plan for daily life, with the aim of targeting activities in a broader policy area than just long-term care prevention. This plan was developed based on suggestions from projects in the area of long-term care prevention described above, as well as from various projects in other areas. Comprehensive plans of local governments in Japan are generally prepared by combining the plans of various departments as separate chapters into a single plan, but this does not lead to an integrated reappraisal of the community and daily life. Therefore, in this project, we attempted to create a single structure for the nine welfare-related administrative plans, and then integrated them holistically into a single comprehensive plan.

In addition, in order to replace Japan’s typical approach to administrative plans, which merely creates a list of “measures that can be implemented at the present time” based on existing administrative resources and past achievements, PONIPONI and OMUTA city decided to include “measures that should be addressed even though no means of implementation have been found at the present time” to create the free space expected to trigger novel co-creation activities.

### 4.7. Entrustment of community comprehensive support centers (2021)

In order to challenge the design of social systems in a more practical manner, we were entrusted with two Community Comprehensive Support Centers, which are community-based, public interest entities. These centers are institutions stipulated in the Long-Term Care Insurance Law and established by local governments for the purpose of comprehensively supporting the improvement of the healthcare and welfare of the older adult by providing comprehensive consultations with the older adult in the community, protecting their rights, creating a community support system, and providing necessary assistance for long-term care. In addition, in response to the recent revision of the long-term care insurance system, OMUTA City has also established a system to actively engage in “community development. Specifically, the center is the first place to receive so-called “in-between problems (system errors)” that occur in the community, and can be said to be the center of a regional network to solve “in-between problems” and promote long-term care. Therefore, it has a great advantage as a center of practice for designing social systems in that it can detect social system deficiencies, draw out collaboration through its network, and work beyond its own domain.

### 4.8. Sign comprehensive cooperation agreement with OMUTA city (2022)

Furthermore, PONI PONI signed a “Collaboration Agreement for the Realization of a Community Coexistence Society” with the city of OMUTA. Its subject is the promotion of the comprehensive plan formulated in 2021. This allows PONI PONI to officially support policy formation in a wide range of areas in conjunction with government departments and to collaborate with stakeholders within and outside the community to realize the vision of the policy. This will help turn around the situation that tends to occur in Japan, where “public matters are left to the government.” The partnership between OMUTA City and PONI PONI, a community-based social system design organization, has officially paved the way for the integrated implementation of policies that have been stove-piped since PONI PONI’s founding by a private intermediary organization. From a different perspective, PONI PONI’s assumption of the planning promotion secretariat has made it possible to promote administrative planning through a collective impact approach.

### 4.9. National model project on housing (2022)

In addition to the Welfare department collaboration, we started collaborating with the housing department of OMUTA City, on the “Model Project on Housing through Cooperation between Welfare and Housing Departments in Local Governments” by the Ministry of Land, Infrastructure, Transport and Tourism. Housing policy is said to be a highly integrated area that is linked to not only welfare but also urban planning and immigration. Naturally, this was one of the themes of the Comprehensive Plan for Health and Welfare, but by focusing on housing, it was possible to gain a detailed understanding of the policy background and conduct a survey of the actual situation in the region. In the process, we further discovered the concept of “substantiating social inclusion”^7^ (Miyamoto, 2017), which expands on Omuta’s new transition concept.

### 4.10. Hosting of the NINGEN societal festival (2022)

**Figure 3:**
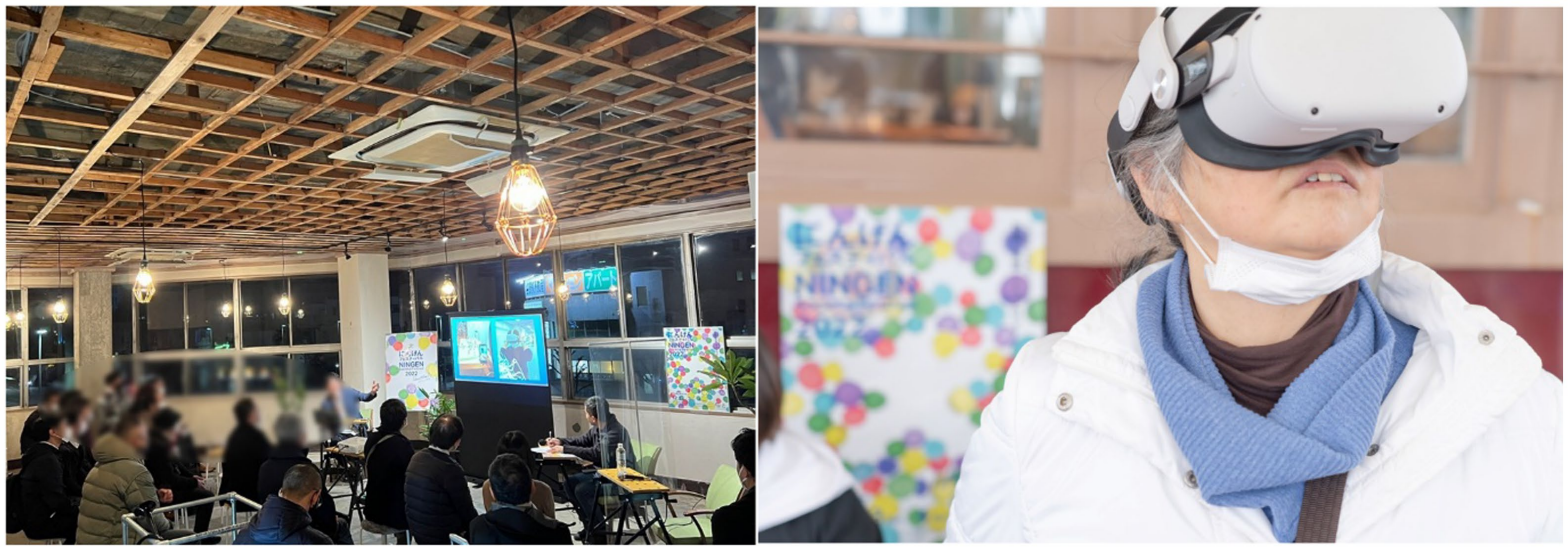
Scene of NINGEN societal festival dialog (left) and technology experience (right).

In 2022, we held the NINGEN Societal Festival (Figure 3) as an opportunity to share and connect with more citizens and other stakeholders within and outside of the region, based on the questions (principles) that emerged and tackled in the various projects (care prevention, housing, education, employment, etc.) that we have worked on with local citizens, local governments, and companies over the past 3 years. The NINGEN Societal Festival was held as an opportunity to share and connect with a larger number of citizens and other stakeholders in the community and beyond.

NINGEN is the Japanese word for Human. The kanji for NINGEN is composed of the kanji for “human” and “aida”/“ma,” which in Buddhist terminology means “between people,” and is used to represent “a place where people live. In the various projects that we have undertaken in Omuta, uncertainty about the meaning of NINGEN has emerged again and again. We have faced up to the questions raised and promoted projects for the future of the community and society. We believe that the concept of NINGEN, which is different from the Western view of human beings, is a universal question that require resolution if we are to tackle social issues common to Japan as well as other countries around the world, where aging and urbanization are also rampant. We then planned the NINGEN Societal Festival, which rethinks the concept of NINGEN.

Fifteen experts and practitioners in various fields engaged in generating advanced and unique questions gathered in Omuta; it provided an opportunity for a large number of participants from within and outside the region, to gather, hold dialogs, and experience technology (Figure 3). This led to efforts to demonstrate new technologies, ways of living, and ways of thinking to children and young people, and to foster a culture in which each citizen feels able to play a leading role for transition to new social systems and to be able to change society.

### 4.11. Project to connect with the community using VR technology (2022)

The aforementioned festival was the starting point for a variety of collaborations, one of which is a project to connect with the community using virtual reality (VR). Together with senior citizens and young people living in Omuta, a 360-degree camera is used to take pictures of favorite and nostalgic places in Omuta, which are then shown via VR systems to residents of nursing care facilities. When they view the memorable places, they will find themselves curious to “go a little deeper” or “touch the things in front of them,” and their bodies and minds will naturally start to move, creating an experience that is unique to VR. For the photographer as well, going out to shoot while imagining the people who will be viewing the images provides an opportunity to reconnect with the “community” and “people” in a different way than in the past.

These series of experiences are connected to the trial of how to implement the questions (philosophy) found in Omuta as technology in society. In fact, a collaborative project has been launched with a company that wants to explore the possibility of “technology to bring out the potential of people,” rather than simply introducing new technology for the sake of management and efficiency.

### 4.12. Model project on long-term care prevention (2023)

With the start of the Model Project on Long-Term Care Prevention by the government in 2023, the practice of structurally rethinking and redesigning existing social systems based on the new principles of “moving from guaranteeing the right to exist (Article 25 of the Constitution) to guaranteeing the right to pursue happiness (Article 13)” and “substantiating social inclusion” is finally being promoted. By making use of hands-on entities such as community comprehensive support centers, which are involved with local citizens on a daily basis, and their local networks, and through collaboration with local merchants who have not been involved in long-term care prevention, and technology providers who can transform the way of long-term care prevention with new technologies, we will be able to accelerate the transformation of local social systems into entities that are not confined to long-term care prevention.

## 5. Social system design methodology

In this chapter, we attempt to systematize our proposal as a general-purpose methodology, using the social system design practice in OMUTA City as a starting point.

### 5.1. Grasping the views of social systems

**Figure 4:**
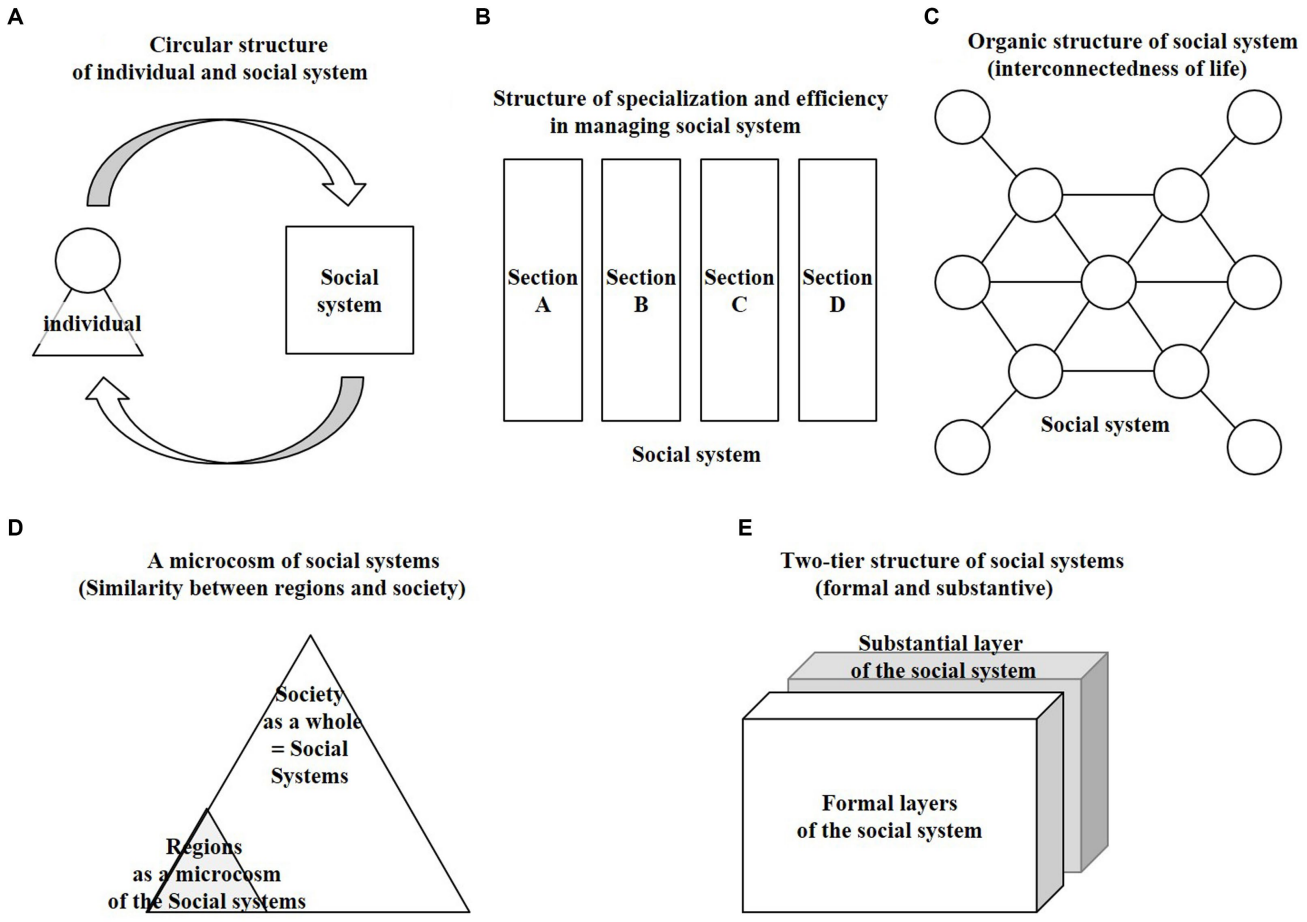
Views of social system structure. **(A)** Circular structure of individual and social system. **(B)** Structure of specialization and efficiency in managing social system. **(C)** Organic structure of social system (interconnectedness of life). **(D)** A microcosm of a social system (Similarity between regions and society). **(E)** Two-tier structure of social systems (formal and substantive).

Formal social systems such as laws and norms do not unilaterally influence people, but function as substantive social system only when people within the system behave in conformity with them (internalization of the system related to footnote No.2), see Figure 4A. Design practitioners are strongly urged to first grasp social systems from the perspective that social systems are cyclically structured.

This makes it necessary to grasp the point that each area of the existing social system has become vertically divided due to specialization to increase efficiency (Figure 4B). On the other hand, people exist as an integrated entity, and each element of daily life is inseparably linked in a network (interrelationship) like an organism (Figure 4C). The discrepancy between the two is often exposed by social issues.

In order to specifically design a social system as a design object, it is necessary to limit the object and make it tangible. Therefore, one option is to target a specific “region” with fixed scope as a microcosm of the social system (Figure 4D).

It is also useful to use “policy” as a pathway to understand and work on the basic framework of the social systems in that region. However, it is important to discern the two-layered structure (formal and substantive) of the social systems. For example, the formal policies can be changed through official procedures, but this alone will not reach the concrete change of social systems. The approach at the “substantive” level, which is the actual implementation of the plan’s principles, requires building relationships and working with government officials and local stakeholders to collaborate in a substantive manner. In most cases, either a formal or substantive approach is taken, but in order to approach both sides (formal and substantive) of a mutually embedded structure for social system transformation, it is essential to obtain formal ostensible standing as well as to implement concrete practices at the substantive level (Figure 4E).

### 5.2. Position of the social system design practitioners

As mentioned in the previous section, the principles of a social system must support both the environment and people’s internal aspects like thoughts and behaviors, as both constitute the system in circulatory manner. Therefore, any entity that seeks to design a social system must internalize while escaping from the circulatory structure and implement a new concept in the existing system. The result being that those activities reconfigure the entire system. As described before, this is the target of an “organization that is both independent and embedded.” Herein lies the basic position and approach of the social system design practitioner.

This is true whether the practitioner is an individual or an organization. In both cases, it is first necessary to free oneself from the functional roles defined by the existing social system. This means creating a situation in which one feels uncomfortable in one’s surroundings as an undefined and contradictory entity. As people will try to pigeonhole the practitioner into an existing role, if the practitioner’s behavior demonstrates compliance, they will become subsumed by the existing system. It is necessary to continue to avoid this while retaining a certain influence on the existing social system. Influence must be both formal and substantive. The formalism acts to create an environment conducive to broad-based movement, while substance contributes to individual, concrete conceptual practices.

As regards concrete practices, the scope of involvement should be unconstrained as much as possible in order to avoid stove-piping (specialization), which is one of the weaknesses of existing social systems. Rather, it is necessary to reconfigure (rearrange) each element of the social system so that new principles can be realized through interaction across a wide range of areas. This also coincides with the breadth of collaboration partners. Social system design practitioners are expected to have a common language and interest in a wide range of areas and sectors, and to take the lead in design.

Financial independence is also important. Receiving compensation for “being of value in the existing social system” can mean being captured by the existing system. In addition, when obtaining funding from a subcontractor’s standpoint, the direction of the design may be strongly constrained by existing philosophies and ideas. In light of these considerations, it is important that funding be indirect, that fair relationships be established as much as possible when making contracts, and that the independent organization should not become too dependent on funds from any one specific entity.

### 5.3. Process model of social system design

**Figure 5:**
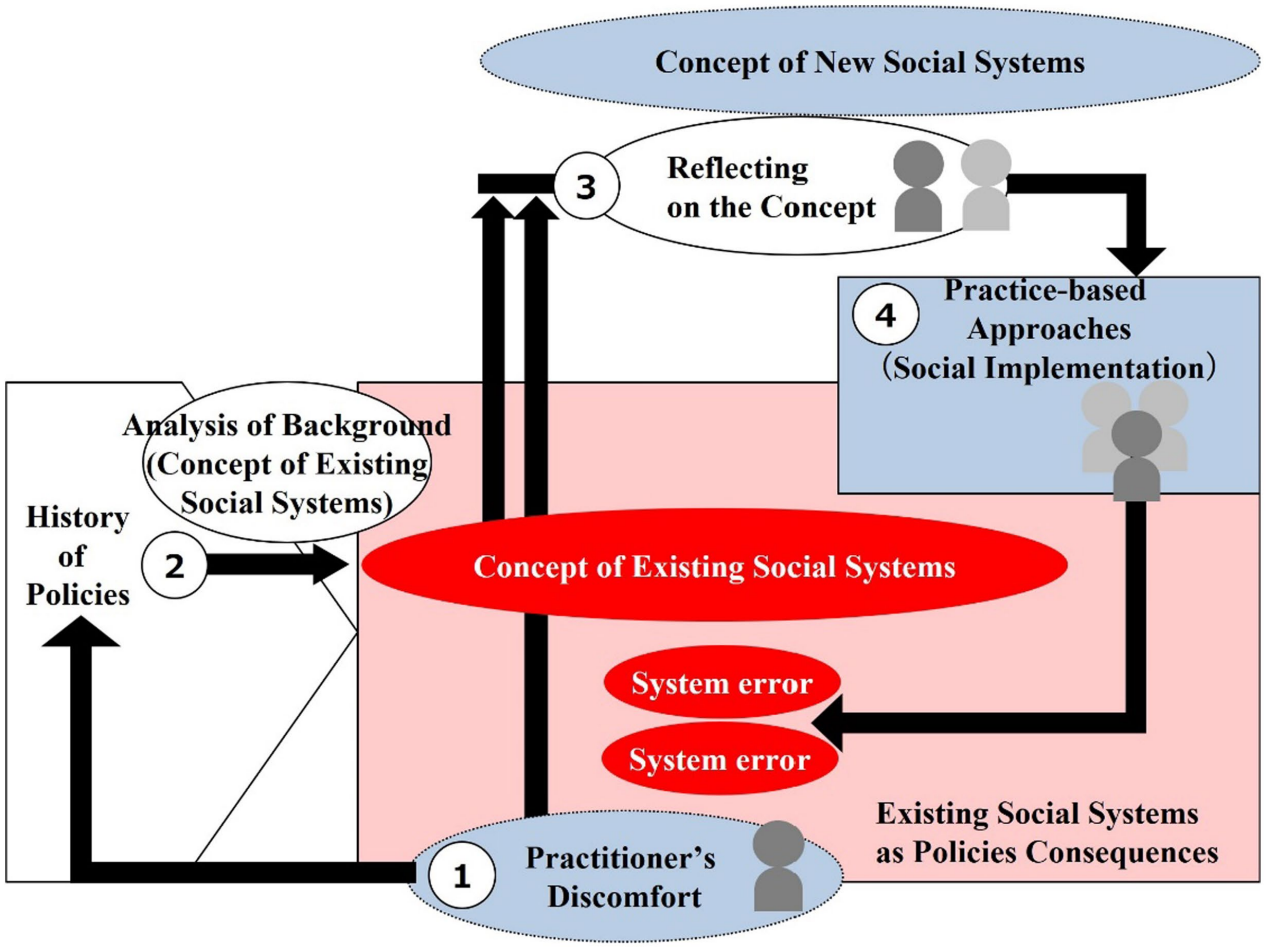
Process of social system design.

This section outlines the process as obtained through practice (Figure 5). It proceeds in an iterated and expanding manner.

**Figure 6:**
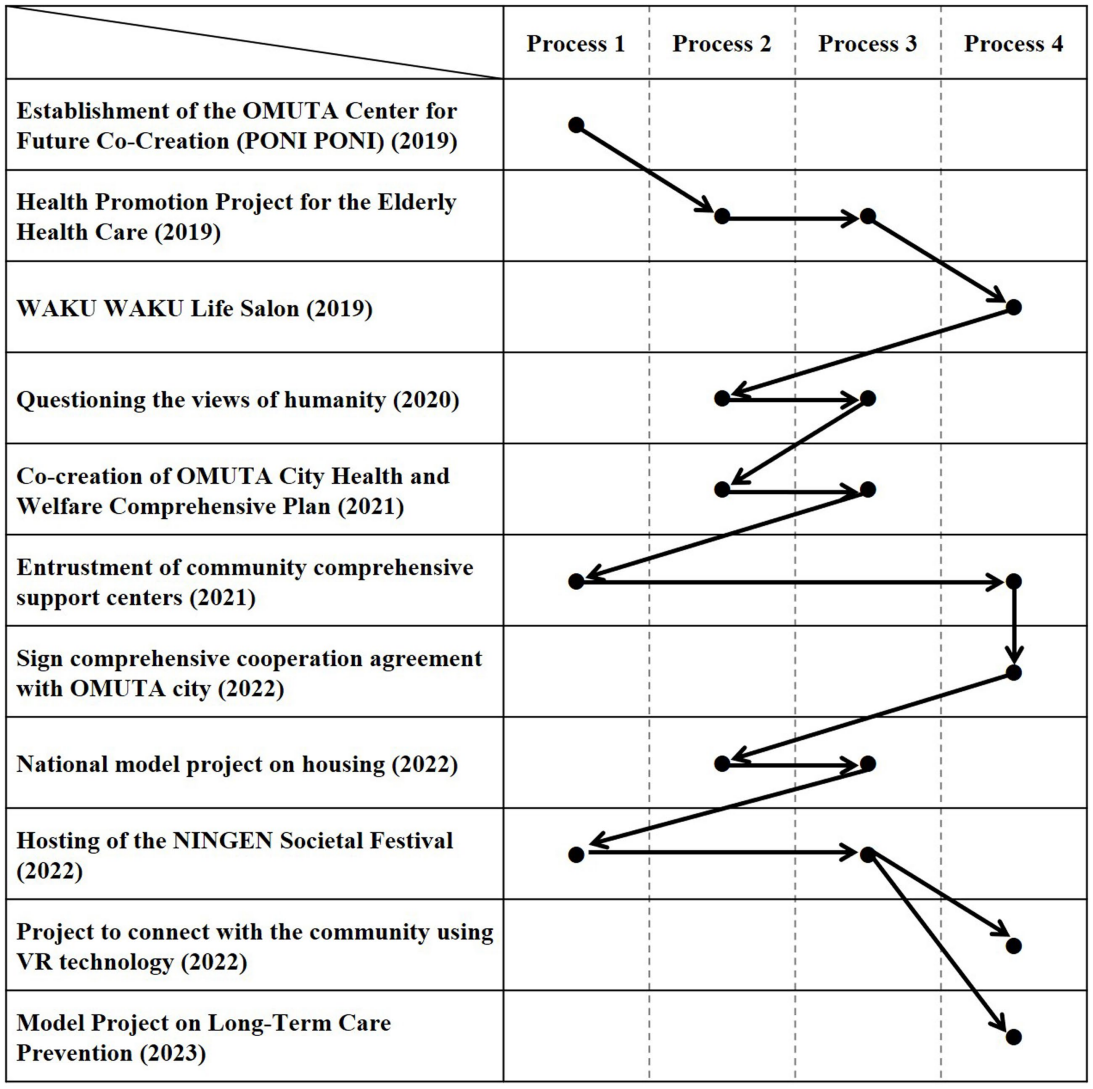
Relationship between process and practices.

Figure 6 shows the relationship between this process and the practices described in the previous section.

#### 5.3.1. Process 1: practitioner’s discomfort

First, it is necessary to create a position in which the “practitioner (individual or team) can be embedded while remaining independent” from the interrelated social system. In this case, the driver for design is the practitioner’s own sense of “discomfort” with the existing social system, as well as the individual’s sense of ownership based on personal experience. Discomfort here means that a person has a sense of being uncomfortable, having his/her freedom inhibited, or having inappropriate involvement with existing social institutions and services. However, it is difficult to cover the wide range of areas involved in designing a social system from just the direct experience of the individuals themselves. Therefore, when working as a team, it is necessary to ensure the diversity of experiences of the members and to take the experiences and positions of others as one’s own. The position of being able to constantly perceive flaws in existing social systems is also a foundation of good design practice.

#### 5.3.2. Process 2: analysis of background (concept of existing social systems)

The social system in front of us exists as if it were self-evident and invisible. However, in many cases, it was implemented at some point. As a clue to this, it is necessary to grasp how the policies were formed, find the structures and principles that created the problems beyond the immediately obvious events, and objectify them. It is important to note that policy intentions can easily change from positive to negative depending on changes in reality. Policy intentions cannot be judged on their content alone. It is necessary to understand the current situation in relation to reality.

#### 5.3.3. Process 3: reflecting on the concept

In order to develop a new social concept to change an existing social system, it is necessary to ask questions about the concept and deepen the dialog. To do so, we need to actively collaborate with experts and practitioners who are challenging society with advanced questions. It is important to open a forum for dialog and questioning, as this will enhance the public nature of our practice and help us find collaborators who are uncomfortable with the existing social system. Furthermore, it is essential to create a circuit that connects these questions to implementation approaches. It is necessary to reflect on the questions in the efforts of design practitioners themselves, as well as to have mechanisms to create new players in the field.

#### 5.3.4. Process 4: practice-based approaches

It is necessary to create practices based on the new principles found, embed them in the existing system, connect them to the existing network, and make them fully functional. Implementing and linking these practices can concretely infuse the existing social system with the new concept and thus give the system a different structure. In other words, this practice means hacking the cycles of individuals and social systems in existing social systems from the inside. It is also important to ensure a network that can permeate the existing social system and expand its functions in a continuous, interrelated, and chain-like manner. It is necessary to pursue not only partial prototyping, but also practices that serve as a pump to spread the concept throughout, so to speak.

## 6. Validation of methodology through case analysis

### 6.1. Target cases

This chapter examines whether the methodology described above is an explanatory model for other practices. The cases to be analyzed are those that attempt to overcome the challenges of the social system design methodology described in Chapter 3, and, once again, are those that satisfy the following two requirements.

Requirement 1: practices that consider the cyclicalities of individuals and social systems.

These are not practices in a free domain that are independent of social systems, but are practices that are intrinsic to current social systems. Moreover, like hacking a computer system with a new program, they are nurtured and modified from within, affecting both individuals and social systems in order to attenuate the discrepancy between existing social systems and daily life.

Requirement 2: practice in collaboration with existing stakeholders.

This is the practice of not only designing systems and services from the top-down in order to transform the social system, but also finding ways to make them function as an entity in the actual living environment (community) through trial and error in collaboration with existing stakeholders.

**Figure 7:**
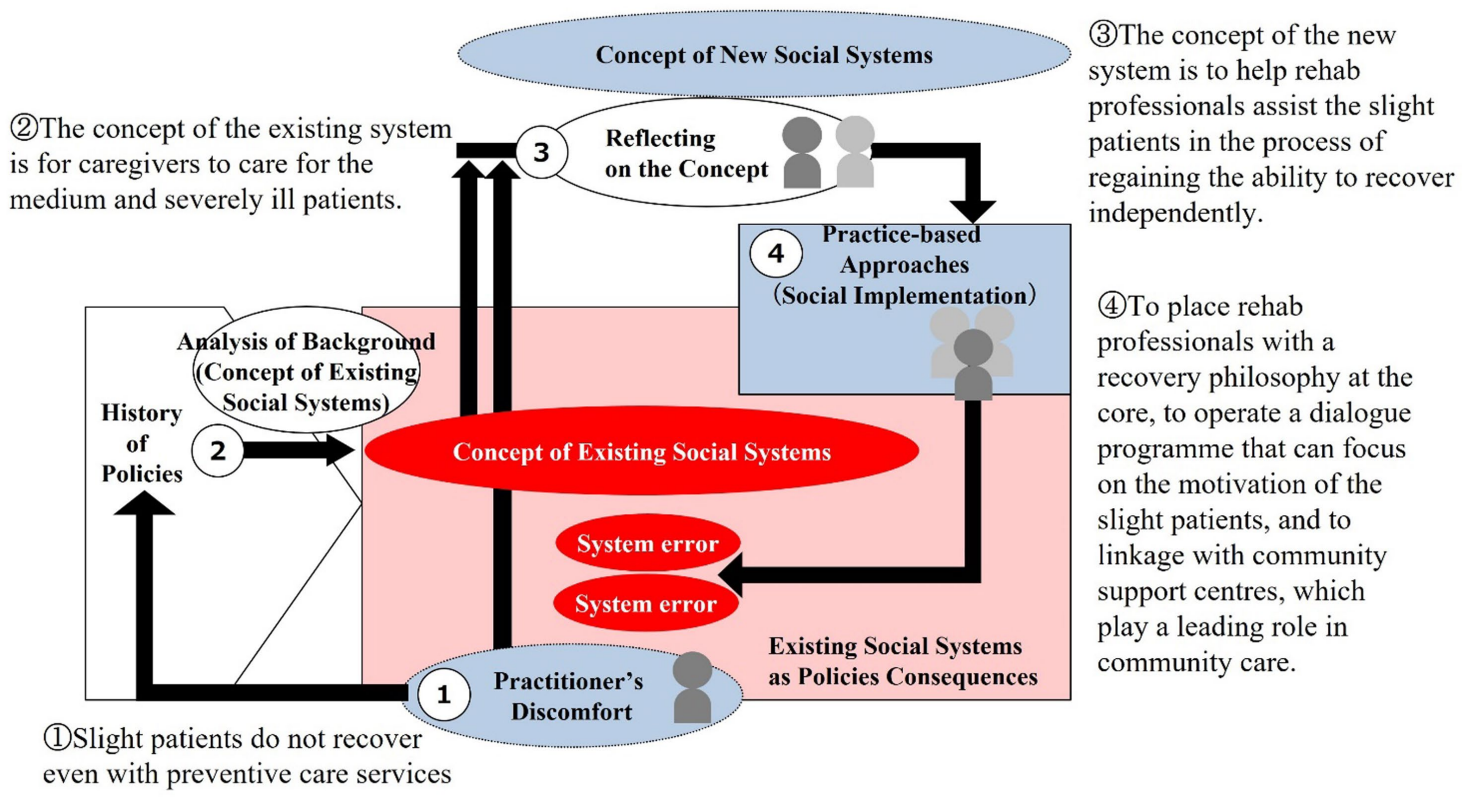
Social system design process for Case 1.

**Figure 8:**
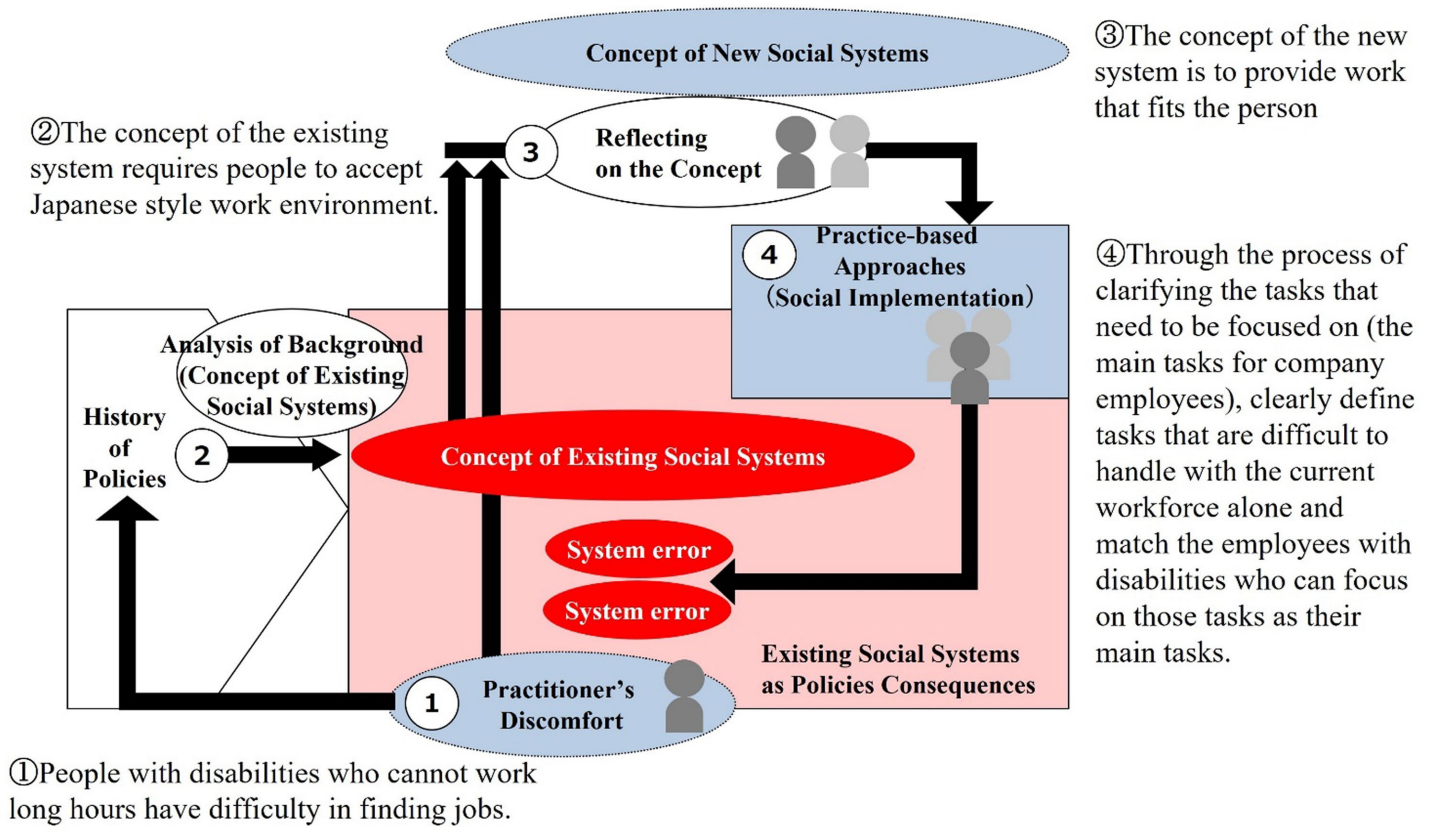
Social system design process for Case 2.

This chapter analyzes two practices in Japan that attempt to meet these requirements from the perspective of social system design methodology. In order to gain a deeper understanding of each practice, we analyzed the logical structure of the practices by reading academic articles written by the practitioners and research reports related to their practices, and then conducted direct interviews with the practitioners to confirm the logic and supplement the information. Case 1 is described in Section 6.2 and its process is illustrated in Figure 7. In the figure, the main points of the case study are described in a way that corresponds to the process from 1 to 4. Similarly, Case 2 is represented in Section 6.3 and Figure 8.

The process model for practice describes building in sequential order from Process 1, finding a new concept in process 3, and then working on the practice in process 4. However, social systems cannot be transformed simply by practices based on new concepts. A social system can be considered to have been transformed when bottom-up practices embody the concept of the system. Therefore, in the case study analysis, after analyzing process 4 practices, we decided to describe the concept of the social system that can be regarded as embodied by these practices in the framework of process 3.

### 6.2. Case 1: practices related to long-term care prevention

The long-term care insurance system has played a central role in Japan’s system of replacing long-term care. The long-term care insurance system is based on the principle of “support for self-reliance” (Ministry of Health, Labour and Welfare [MHLW], 1997). With the aim of increasing the effectiveness of the system, the Long-Term Care Insurance Law was revised in 2005 to amend the preventive benefits and establish community support programs, with the aim of preventing the need for more intensive long-term care. After revisions in 2008 and 2011, the 2014 revision launched the Comprehensive Project for Long-Term Care Prevention and Daily Life Support, along with the enhancement of community support services.

#### 6.2.1. Process 1: practitioner’s discomfort

However, as of the end of March 2020, the number of light patients (those requiring support 1 and 2 and those requiring long-term care 1) will exceed 3.2 million, 3.2 times the number as of the end of March 2001, and the increase in the number of persons requiring light patient care is significant (Ministry of Health, Labour and Welfare [MHLW], 2021). On the other hand, the various services provided by the comprehensive project for long-term care prevention and daily life support are not being fully utilized (Ministry of Health, Labour and Welfare [MHLW], 2022b). This can be considered to mean that the system surrounding long-term care prevention is not functioning effectively, that system-errors have occurred. This means that the system is following principles different from those set forth in the law.

In response to this situation, Hattori and others redefine “independence” in “support for independence” by clarifying “reduction or prevention of deterioration of the state of long-term care required, etc.” in Article 2 of the Long-Term Care Insurance Law as “recovery to a state where long-term care is no longer required (recovery to a state where it is no longer necessary to use the system)” (International Longevity Center Japan Longevity Society Development Center, 2019), and to realize this, the short-term intensive preventive services implemented in the comprehensive project for long-term care prevention and daily life support are positioned as a key measure to shift the philosophy. Efforts are being made to put them into practice so that they can provide effective support.

#### 6.2.2. Process 2: analysis of background (concept of existing social systems)

Under the long-term care insurance system, the maximum amount of benefits from the long-term care insurance system (maximum amount of benefits) is determined for each level of care required, and a change in the level of care required due to improvement means a decrease in income for the service provider. This means that there is a disincentive to improve the level of care required. Incentives for service providers to support independence have long been the subject of discussion at the Subcommittee on Long-Term Care Benefit Expenses of the Social Security Council, but at present, evaluation of “maintenance and improvement of the level of care required” has been implemented only for some services (Ministry of Health, Labour and Welfare [MHLW], 2017). Furthermore, there is no limit to the period of time that services can be used, except for short-term intensive preventive services, making it easier to sustain their use.

On the other hand, the comprehensive project for care prevention and daily life support has not been implemented uniformly throughout the country, which had been the norm until then, but is designed as a system that can be implemented according to the actual conditions of each municipality. This has resulted in a large difference between municipalities that are willing to implement the program and those that are not, and the fact that the approach to implementing the program differs from that of the past has also become a hurdle, which has prevented the program from spreading as a whole.

In other words, contrary to the philosophy set forth in the Long-Term Care Insurance Law, the long-term care insurance system has become a mechanism that does not provide effective incentives for “recovery to a state where long-term care is no longer required. In addition, the comprehensive project for long-term care prevention and daily life support challenged the willingness and policy understanding of the municipalities that were the implementing entities. As a result, it can be said that the operation of the system was driven by the principle of promoting “continuous use of the system,” rather than “recovery to a state where long-term care is no longer required.

#### 6.2.3. Process 4: practice-based approaches

In contrast, the practice of Hattori and others positions and utilizes the short-term intensive preventive services implemented in the comprehensive project as a core measure of long-term care prevention and daily life support.

First, they recommend that municipalities establish a process whereby basically all persons eligible for the project^8^ or those requiring support 1 and 2 who use the long-term care insurance system for the first time, with some exceptions, first use the short-term intensive preventive services. This creates a new preliminary step in the operational process of the existing long-term care insurance system, creating an area where a new philosophy can be easily realized locally, while still having links to the latter process and existing stakeholders.

Second, they have developed and implemented a dialog program that focuses on the motivation of the light patient. This is a support program centered on dialogs that draw out the motivation of light patients, in which they envision the life they would like to regain after improvement, and think together about goals and initiatives in their daily lives outside of the days when services are provided (used). Service use is basically limited to 3 months, or 6 months under special circumstances.

In the program, self-management is the foundation, and the philosophy of “recovery to a state where nursing care is unnecessary” is implemented in the form of “support for doing” (The Dia Foundation for Research on Ageing Societies, 2018). In order to achieve this, rehabilitation professionals (occupational therapists, physical therapists, etc.) accompany the light patients after full assessment of their abilities. Although rehabilitation professionals have recovery (rehabilitate) rather than nursing care (care) as their core expertise, they have not had a central role in long-term care prevention measures in the long-term care insurance law. Therefore, it can be seen that they have gained a position where they can move with a degree of freedom toward the realization of their philosophy, while being embedded in the existing system for short-term intensive preventive services.

In addition, the short-term intensive preventive care services are basically provided by the community comprehensive support centers operated by the municipalities. Since the role of these centers is to provide guidance (comprehensive and continuous care management) to the in-home care support offices that will collaborate with them in providing care management for care prevention in the community, they will serve as a driver to expand the new philosophy.

On top of this, Hattori and others. Propose that short-term intensive preventive services, community rehabilitation activity support projects, community care meetings, and lifestyle support coordinators, measures that are related to long-term care prevention but have been difficult to link, organically, be linked and operated in an integrated manner (Mitsubishi UFJ Research and Consulting, 2019). This encourages synchronized advances in multiple measures for long-term care prevention and helps municipal officials understand the system.

#### 6.2.4. Process 3: reflecting on the concept

The practice of Hattori and others is to redefine the philosophy of “support for self-reliance,” which has been the legal phrase of long-term care insurance since the establishment of the system, as “recovery to a state where long-term care is no longer needed,” and to make it a concrete goal of short-term intensive preventive services. The idea is to make the philosophy permeate the existing system and to reconfigure the entire system together with the philosophy by making it fully functional.

New professionals (occupational therapists, physical therapists, etc.), whose core expertise is in recovery (rehabilitation), will play a central role in implementing the services with limitations on the period of use, setting “recovery to a state where nursing care is no longer required” as a specific goal (outcome). Since the municipality designates the providers, there is no excessive competition among providers, and the system can be operated as a system that offers stable profits. Moreover, involving the community comprehensive support centers that will spread the philosophy, and by operating in combination with related systems, it becomes possible to change the entire system while hacking the existing system.

Furthermore, by creating one successful case study in each prefecture, Hattori and others hope to encourage municipalities, which tend to take a wait-and-see approach, to stimulate a sense of crisis in the municipalities by creating a situation where neighboring municipalities are working on good mechanisms, and to spread the care prevention system based on the new philosophy throughout the country.

The efforts of Hattori and others should truly be a good example of social system design, where the project hacks into and changes existing social systems.

### 6.3. Case 2: practices related to employment of people with disabilities

The Convention on the Rights of Persons with Disabilities, adopted by the United Nations in 2006 and ratified by Japan in 2014, calls for the “prohibition of discrimination on the basis of disability” and “reasonable accommodation” in various policy areas such as education and employment. The Convention on the Rights of Persons with Disabilities advocates the principle of “inclusive.” Article 3 of the Convention stipulates “full and effective participation and inclusion in society” as one of its general principles. Regarding labor and employment, Article 27 stipulates the right of persons with disabilities to work in an “open, inclusive and accessible” labor market and working environment. Japan’s system of employment of persons with disabilities, which includes a system that legally obliges companies to employ persons with disabilities (e.g., an employment ratio system for persons with disabilities), has been developed in conjunction with the ratification of the Convention. At the same time, the systems of support for transition to employment and continuous employment support as welfare services for persons with disabilities have also been revised.

The employment rate system for persons with disabilities is a system that requires a certain percentage of workers employed by a company to be persons with disabilities.^9^ There, 1.0 person is counted for 30 h or more per week (2 persons for severe physical or intellectual disabilities), and 0.5 persons (1 person for severe physical or intellectual disabilities) are counted for short-time workers who work 20 to 30 h per week. As a special exception to this system, if a company’s employer establishes a subsidiary that makes special arrangements for persons with disabilities and meets certain requirements (Ministry of Health, Labour and Welfare [MHLW], 1960), the “special subsidiary” system allows workers employed by the subsidiary to be counted in the actual employment rate as if they were employed by the parent company or the entire corporate group.

On the other hand, as welfare services, persons with disabilities who wish to work at general companies can receive support for employment at labor transition support facilities. There are also two types of welfare employment for persons with disabilities who find it difficult to work at general companies: Type A continuous employment support pays wages (above the minimum wage) for labor, while Type B provides a wage (national average: about 16,000 JPN).

#### 6.3.1. Process 1: practitioner’s discomfort

The total number of persons with physical, intellectual, and mental disabilities is approximately 9.65 mil., of which approximately 3.77 mil. Are homebound persons between the ages of 18 and 65 (Ministry of Health, Labour and Welfare [MHLW], 2022c). As of 2022, there were approximately 614 thou. Persons with disabilities employed in the private sector (Ministry of Health, Labour and Welfare [MHLW], 2022a). The breakdown is as follows; The total number of special-purpose subsidiaries is 579, and the number of persons with disabilities employed is approximately 44 thou. The number of persons with disabilities employed by public organizations, etc. (national, prefectural, municipal, board of education, and independent administrative institutions) is 83 thou. On the other hand, the number of users of welfare employment was 375 thou. (Ministry of Health, Labour and Welfare [MHLW], 2009). Looking at this from the perspective of the employment of persons with disabilities, less than half (48.3%) of the companies meet the current legal employment rate (2.3%) (Ministry of Health, Labour and Welfare [MHLW], 2022a).^10^ In addition, the rate of transition to general employment at labor transition support facilities remained at 54.7% (in FY2019) and in the case of welfare-type employment, the transition rate to general employment was 25.1% for Type A and 13.2% for Type B (Ministry of Health, Labour and Welfare [MHLW], 2022c).

Although the number of persons with disabilities employed in the private sector continues to increase, this number is not as large as the actual number of persons with disabilities, and the system surrounding the employment of persons with disabilities is not fully functioning.^11^ In 2022, the UN Committee on the Rights of Persons with Disabilities, in its recommendations to Japan, calls for a stronger transition from “protected workshops and employment-related welfare services” to “open labor markets in the private and public sectors” and “equal remuneration for work of equal value in an inclusive working environment.^12^ The reality is that people with disabilities who have difficulty working more than the 20 h required by the legal employment rate are not expected to work in companies in the first place.

These situations indicate that the principles of the Convention on the Rights of Persons with Disabilities are not functioning as the principles of the system for employment of persons with disabilities in Japan. In response, Kondo and others. Are focusing on employment opportunities of less than 20 h, by creating and putting into practice the “ultra-short-time employment model” as a system implementation of the principles.

#### 6.3.2. Process 2: analysis of background (concept of existing social systems)

Kondo points out that traditional Japanese employment practices are behind the problems in the employment of people with disabilities in Japan. According to Kondo, Japanese employment practices are based on the premise of “permanent employment,” in which new graduates are hired and employed full-time by a single company for an indefinite period of time, with a seniority-based wage system that provides security of livelihood with long-term prospects. This practice, known as “membership employment” (Hamaguchi, 2011), is also the premise of Japan’s social security system, whereby people are connected to the unemployment insurance safety net in addition to their wage security through membership in a company.^13^

However, according to Kondo, the “need to work long hours” and the “lack of job definition at the time of hiring^14^“in this uniquely Japanese employment practice are the factors that exclude people with disabilities. Kondo points out that Japanese employment practices require all employees to “orient and clarify unclear duties in accordance with the changing mission of the company” and, as the basis for this, to have “the ability to communicate with others at a high level” (Kondo, 2016). Thus, it can be said that the conventional Japanese employment system operates based on a philosophy that uniformly emphasizes human resources who can fulfill any duties in a flexible manner.

Regarding this current situation, Kondo evaluates the significance of the employment rate system for persons with disabilities as “an effort to increase the number of people certified as having a disability in the form of holding a disability certificate in a form of employment similar to regular employment” However, he points out that “strong institutional backup to encourage the employment of people with disabilities” in line with Japanese employment practices “has, on the contrary, created a situation where it has become a barrier that makes it difficult for them to enter the regular workplace” (Kondo, 2016). In this regard, Kondo is also critical of special-purpose subsidiaries and businesses that utilize legal employment quotas for people with disabilities, because while they may achieve the employment rate of people with disabilities in terms of numbers, they may actually promote a situation in which people with disabilities and able-bodied people are separated in the actual workplace.

#### 6.3.3. Process 4: practice-based approaches

In order to solve this problem, it is necessary to critically rethink Japanese-style employment practices (the philosophy of the current system) and create a situation in which people with disabilities can work alongside able-bodied people in regular workplaces. To this end, companies that employ people with disabilities must improve their working environments to be more inclusive, rather than unilaterally conforming to the current system of employment. Kondo and others call this the “ultra-short-time employment model” (Kondo, 2020)^15^ and are working on the corresponding practices. It consists of “a way of working that allows people to have a role in the regular workplace, even if it is only for a few minutes or hours a week, a support system in the community to realize such a way of working,” and “technology to create an internal work and employment environment.

When introducing the ultra-short-time employment program, Kondo and others ask companies to forget about employment of people with disabilities for a moment and focus on the work of a specific staff member working at the company. Then, while reaffirming the values and ideal forms that they want the staff to realize, they break down the work into (1) the essential tasks that the staff should perform, (2) peripheral tasks that the staff should perform, and (3) tasks that do not necessarily have to be performed by the staff but are required to be performed in the workplace. (3) Tasks that do not necessarily need to be carried out by the staff member in question, but which must be handled in the workplace. After clarifying the (3) operations broken down here as jobs, the next goal is to recruit, hire, and retain people who can handle these specific operations (jobs). At this stage of recruitment, the employment support system for people with disabilities, including welfare-type employment, is actively utilized.

Here, Kondo and others, do not consider the Japanese-style employment practice of undefined duties as causing problems only for persons with disabilities, but rather see it as a problem that companies generally face, and take a new approach by reorganizing the division and assignment of work in the workplace. What is important is that the specific tasks in (3) are not tasks that have been specifically carved out for people with disabilities, nor are they tasks that anyone can do, but are clearly defined as tasks that are necessary for the workplace but difficult for the workplace staff to handle alone. That work is defined as work that is necessary for the workplace but that cannot be handled by the workplace staff alone. In addition, the emphasis in the very-short-time employment system is on “not asking the person who performs the job to do anything other than what is necessary for the job.” By establishing each job as a job in both definition and practice, the workplace is reorganized into a workplace that consists solely of each individual’s original job, and everyone stands on the same level. By creating such a fair structure in the workplace, the ultra-short-time employment model is expected to substantiate the inclusive philosophy of “working together.” Such an ultra-short-time employment model has produced many cases in which users of Type B continuous employment support, a type of welfare-type employment for people with disabilities who find it difficult to work in general companies^16^ (Kondo, 2020), have been employed. This fact sharply forces us to reexamine the meaning of “being able to work in a general company.

#### 6.3.4. Process 3: reflecting on the concept

Japanese-style employment practices today are thought to be a factor that alienates not only the disabled but also women, the older adult, and other diverse work styles, as well as being a factor in health problems such as overwork and depression caused by excessive concentration of duties on those who are skilled in progress management and communication skills. This was thought to be appropriate when healthy adult males could be assumed to constitute the labor force, but today, when a diverse workforce including disabled persons, women, and the older adult exists, it is also pointed out that this has led to a decline in the vitality of society (Hamaguchi, 2011).

The ultra-short-time employment model has been implemented in local shopping areas in Kobe City and in small and medium-sized enterprises (SMEs) with less than 50 employees in Kawasaki City. Kondo says that the model has been realized in these regions. According to Kondo, since the ultra-short-time employment model is job-type employment^17^ that does not presuppose continued employment, it is necessary to have a function that supports the mobility of workers who can work for another company by drawing on their career even after their employment ends because the tasks no longer exist. Coordination and networking for this function are currently required by the existing social employment system in the region. In this regard, Kondo also says that the ultra-short-time employment model “is not a conventional model in which a single company employs one person with disabilities for a long period of time and continues to guarantee his/her livelihood, but a model that is closer to the idea of ‘employment in the community’“(Kondo, 2020).

Here, the existing social work system will be actively reimagined in the region as necessary for the regional implementation of the ultra-short-time employment model, helping to open the employment of people with disabilities from “protected welfare services” to an “open labor market. In this respect, the ultra-short-time employment model does not conclude the story of the workplace, but is considered to substantiate the idea of inclusiveness by hacking the existing system in the regional phase.^18^

## 7. Discussion

Case 1 expresses the fact that top-down philosophical concepts alone are not enough to achieve social system transformation. It is difficult to rewrite the concept of a system given the cyclical nature of individuals and social systems simply by renewing administrative systems. Therefore, the Social System Design Methodology recommends working on the ground and hacking the existing social system with practices based on the new system concept. Hattori worked through a series of practices, such as placing rehabilitation professionals with the philosophy of recovery at the core of their practice, operating a dialog program that can focus on motivating the minorities, and letting the community comprehensive support centers play a leading role in the care of the community. These multi-layered, intertwined and complex practices in the field have created a situation in which a substantially new social system’s concept has emerged. As a result, there are signs of an expanding social system in which the light patients are becoming motivated and thus recovering.

Case study 2 shows that even initiatives that appear to be improving toward a new concept will not eliminate barriers to genuine employment for people with disabilities unless the essential concept is transformed. Kondo finds that the concept of the existing system is influenced by unique Japanese employment practices, among which the need for long working hours and the lack of job definition at the time of recruitment are the main disincentives for people with disabilities to work. It is then oriented toward a substantially new concept of the employment system by overlaying practices such as the process of finding work from the standpoint of realization of the company’s mission, making rules for disabled people to work at the company site without anxiety, and building model cases that can be used for the existing legal employment rate. This has created a new system that enables many people to work with their own strength in their community.

Through the analysis of these case studies, it has been shown that the social system design methodology can be applied to cases where social systems are being transformed in concrete ways.

## 8. Contributions and limitations

The contributions of this paper are the development of a social system design methodology based on the analysis of a series of practices developed in Omuta, Japan, and the confirmation of the generality of the proposed methodology through two case studies examining care prevention and employment issues for people with disabilities.

This methodology provides insights into perspectives that have not been described in previous academic theories. The challenge with transition management has been that the agenda is considered by multiple stakeholders, but the specific practices that follow do not proceed. In response, the Social System Design Methodology proposes a methodology not only for developing agendas and policies that express concepts, but also for people to hack the system in the field of practice to make the concepts permeate the system.

Also, the challenge for the Urban Living Lab was that it had not established a methodology for setting issues in the complex and intertwined urban dimension, although it was equipped with methods to promote the engaged participation of citizens. In response, the Social System Design Methodology proposes an approach to problem-setting that is oriented toward system transformation at the urban level through an analysis of the policy context starting from Practitioner’s (citizens’) discomfort and a dialog that clarifies the concept of a new social system.

The Social System Design Methodology showed policy makers and practitioners in the field that it is difficult to transform to a new concept if policy makers and practitioners in the field work independently of each other. It then proposes how policy makers and frontline practitioners should co-create activities to transform social systems. Specifically, concept-oriented practices cannot emerge in the field simply by being included in administrative plans and agendas as language and text. On the other hand, concept-oriented practices in the field alone will not spread the concept to society, influenced by the structure of the existing system. In order to overcome these problems, it is suggested that the project be initiated with the designer’s (policy maker’s or practitioner’s) discomfort as the starting point, and that the analysis of policy background and dialog of questions be conducted in the early stages of the project, and that a team that shares discomfort be launched in the process.

Based on these implications, this study’s contributions include overcoming the situation where traditional living lab projects have tended to be partial solutions to specific social problems. The practices of Omuta Living Lab follow this methodology, so the co-creation projects that are launched here consist of practices on the ground in the community, linked to the concept of a new social system. As a result, projects are being created that propose models for future housing, learning, mobility and care prevention, while also staying close to the issues at hand for citizens, and this is where municipal plans and new business development of companies are linked to these activities. In addition, attracted by the concepts challenged by these practical models, researchers and practitioners from diverse fields are visiting Omuta to engage in dialogs or projects.

In other words, Omuta Living Lab’s practice is not a living lab that tackles just the problems at hand, nor is it a living lab that explores just new futures (like speculative design (Anthony and Fiona, 2013) or future center (Dvir et al., 2006)), but a living lab that creates the future by identifying the root causes connected with the problems at hand and transforming the problem structure (social system). It could be called a living lab that creates innovation in meaning (Verganti, 2009) for the future.

On the other hand, it is a limitation of this study that this legal theory is built on Japanese practices and cases. Citizenship and the relationship between citizens and society in Japan are contextualized differently than in other countries, and the meaning of the concepts of subjectivity and autonomy are different. Whether social system design methodology is applicable to countries in East Asia with similar human perspectives as Japan, and whether it is applicable to countries in the world different from them, is a subject for further study and discussion.

## 9. Conclusion

This paper provids a theoretical overview of the difficulties posed by social system transformation and a design logic to overcome these difficulties. It also presents concrete time-series examples to elucidate the practice of the approach, from which a general-purpose social system design methodology was derived. The applicability of the methodology was also tested by analyzing two good examples of social system transformation based on a process model of social system design.

In order to develop this methodology into something more versatile and useful, it is necessary to further elaborate its contents and clarify the leadership required of practitioner’s (designer’s) and the nature of actual environments. Furthermore, this methodology should never be seen as complete. It is important that the methodology offer “continuous change” in order to respond to major shifts in new values such as the SDGs, based on the fact that modern social systems have characteristics that tend to move away from an integrated way of being and living.

## Data availability statement

The original contributions presented in the study are included in the article/supplementary material, further inquiries can be directed to the corresponding author.

## Author contributions

All authors were involved in the establishment of the Center for Person-Centered Ningen, Omuta (PONI PONI). All authors involved in the practice of social problem solving projects and the description of their practices.

## Footnotes

^1^According to Inaba, while social movements such as the Democracy Movement and Occupy Movement were revitalized worldwide in the 2000s, such movements were not so vibrant in Japan and non-protest-oriented movements attracted attention. Inaba acknowledges that social movements have become a place for minorities as described in the body of this paper; she also pointed out the need to find new political possibilities.

^2^“Mutuality and circularity between the individual and society” refers to the way in which our decisions and behaviors constitute the social system, and at the same time, the constituted social system also defines our decisions and behaviors. For example, let us consider the automobile traffic system. We can imagine it as a system of physical environment and rules, including traffic infrastructure such as roads, pedestrian crossings, and traffic signals, as well as traffic rules such as “stop at red lights” and “speed limit of 50 km/h.” However, no matter how elaborately designed the traffic infrastructure and rules are, they alone will not make the traffic system function. It must be designed in such a way that drivers and pedestrians behave in accordance with the rules. Therefore, in order for a transportation system to work, while infrastructure and systems are being developed, people must be trained to control their bodies, for example, to “stop at red lights,” and systems are being implemented in schools and homes to educate people to behave in accordance with the rules. The fact that people are encouraged to behave according to the rules is called “Mutuality and circularity between the individual and society. Such understandings are based on M. Foucault’s theory of power [especially the discipline model (Foucault, 1975)]. This paper, however, while following this Foucauldian understanding, explores a design methodology that differs from Foucault’s “archeology” and “genealogy” approaches.

^3^Manzini, an expert on social innovation, points out that modern society requires deep and significant systemic change (on the same level as the prior transition from feudal to modern society), and that what does not touch the roots of the system will not help in systemic transformation (Manzini, 2019).

^4^In January 2005, Omuta City issued the “Declaration for Creating a City to Live with People with Dementia” in order to make it known throughout Japan that the entire city will support people with dementia and their families through cooperation among welfare, medical care, nursing agencies, the community, and government [https://www.city.omuta.lg.jp/hpKiji/pub/detail.aspx?c_id=5&id=6652&class_set_id=1&class_id=136 (Accessed March 1, 2023)].

^5^PONI PONI’s website (https://poniponi.or.jp/) and the ‘Questions and Dialog’ media (https://dialogue-eureka.jp/) jointly run by PONI PONI and the Co-Designing Institute for Polyphonic Society (Accessed March 1, 2023).

^6^The modern subject is a human being who organizes his or her inner self to conform to various social institutions, and whose voluntary behavior contributes to the maintenance and strengthening of those institutions. It is also a view of human beings that regards such a way of being as “normal.” Michel Foucault critically examined this view of human beings relative to the unique context of Western modernity in which it was established. Foucault sees “what is called ‘human being’ in the 19th and 20th centuries” as a combination of the “legal individual” as an ideological subject of rights and the “disciplinary individual” as a useful body obedient to normative power, and criticizes its ideological nature as “that illusion and reality called ‘human’ being.” (Foucault, 2003, p. 60).

^7^Miyamoto cites the criticism of Young (1999), author of “Bulimic Society,” that “repeatedly promoting social inclusion while society remains exclusionary will ultimately promote exclusion. He then points out that while it is good to include all excluded people as members of society, it is a logical contradiction to include people in an exclusionary or self-help society, and that social inclusion cannot be practically realized unless society itself changes.

^8^Officially, “persons eligible for long-term care prevention and lifestyle support service projects. This refers to those who use only the services provided by the Long-Term Care Prevention and Lifestyle Support Services Project and who fall under the category of those who have completed the basic checklist.

^9^Companies that have not achieved the legally mandated employment rate must pay the government a payment corresponding to the shortfall, while companies that have employed more people with disabilities than the statutory employment rate will receive an adjustment payment from the government corresponding to the excess.

^10^The actual employment rate in the private sector is 2.25%.

^11^In this regard, it has long been pointed out that the challenge in employment for people with mental disabilities is not “getting a job” but “getting a job and continuing to work” because the number of people with mental disabilities employed is small in comparison, although statistically the number of people with mental disabilities employed is very high (Kurachi, 2014).

^12^Committee on the Rights of Persons with Disabilities (2022). Concluding observations on the initial report of Japan. The original text of the quoted passage is below. The committee also made a total of four other recommendations regarding employment, including the implementation of reasonable accommodation.
*58. The Committee recommends that, in line with target 8.5 of the Sustainable Development Goals, the State party:*

*(a) Enhance efforts to speed up the transition of persons with disabilities from sheltered workshops and employment-related welfare services to the open labour market in the private and public sectors, with equal remuneration for work of equal value, in an inclusive work environment;*

^13^These forms of employment created high efficiency during the postwar high-growth period through long working hours and transfers, and were suitable for a time when only able-bodied adult males were assumed to be in the labor force (Hamaguchi, 2011).

^14^If a job definition is exchanged, it becomes easier to “undergo appropriate modification and adjustment outside of the essential job duties = receive reasonable accommodation.” But the fact that it is common for this not to be defined, Kondo says, suggests that there is a background where reasonable accommodation is easily denied (Kondo, 2016).

^15^The characteristics of this model can be summarized as follows: (1) job descriptions are clearly defined before hiring, (2) employees work in specific defined jobs for very short hours, (3) employees are not required to do anything other than what is essentially necessary to perform their jobs, (4) employees work together in the same workplace, (5) there are regional systems to create very short-time employment, and (6) the total employment rate is independently calculated. In introducing the ultra-short-time employment model, the following steps are taken: (1) sharing the same philosophy, (2) defining the job, (3) hiring, (4) employment, and (5) retention [Kobe City (Disability Support Division, Welfare Bureau), 2021, Very Short-Time Employment Case Studies, https://www.city.kobe.lg.jp/documents/46683/jireisyuu.pdf (Accessed March. 1, 2023)].

^16^Many people with mental disabilities are working in the ultra-short-time employment model, and many of them are users of Type B continuous employment support (Kondo, 2020).

^17^According to Hamaguchi, “all countries except Japan are job-based societies,” but he points out that all countries except the U.S. have restrictions on dismissal, and the common belief in Japan that job-based employment means free dismissal is a misunderstanding (Hamaguchi, 2011, p. 110). How job-type employment should be in Japan is a separate issue to be considered.

^18^While implementing the ultra-short-time employment model, Kondo and others have also continued to lobby the government to include employment of less than 20 h in the statutory employment rate. In 2022, the Law for Employment Promotion of Persons with Disabilities is expected to be amended to allow employers to calculate the employment rate for persons with mental disabilities, persons with severe physical disabilities, and persons with severe intellectual disabilities who work more than 10 h but less than 20 h as a special case.
